# Preparation and Characterization of Ferulic Acid Hydrogel and Its Application as a Local Drug Delivery Agent in Periodontitis

**DOI:** 10.7759/cureus.60534

**Published:** 2024-05-18

**Authors:** Shubhangini Chatterjee, Arvina Rajasekar

**Affiliations:** 1 Periodontics, Saveetha Dental College and Hospitals, Saveetha Institute of Medical and Technical Sciences, Saveetha University, Chennai, IND

**Keywords:** haemolysis, biocompatibility, local drug delivery, periodontitis, ferulic acid

## Abstract

Introduction

Periodontitis, a persistent inflammatory condition, impacts the tissues supporting teeth. Beyond mechanically eradicating the biofilm, additional host-modulating agents can aid in the treatment of periodontitis. Among these, gels are a very popular choice for use in the field of dentistry as these systems boast high biocompatibility and bioadhesiveness. These qualities make them easily administered and fabricated. They are typically placed into the periodontal site via wide-port needle syringes. Many investigations have demonstrated that hydrogels possess the ability for controlled drug release and aid in periodontal wound healing. Hence, this study aimed to develop a ferulic acid hydrogel and assess its effectiveness for managing periodontitis.

Materials and methods

Ferulic acid hydrogel was prepared followed by haemolysis assay and biocompatibility assay. After the in vitro analysis, a clinical trial was conducted: 20 patients were divided into Group A (comprising patients in whom scaling and root planing (SRP) was done) and Group B (comprising patients in whom SRP along with hydrogel application was done). Each patient's pocket depth (PD), clinical attachment loss (CAL), gingival index (GI), and plaque index (PI) were recorded at baseline and at three months. Intergroup and intragroup comparisons of the parameters were made.

Results

Ferulic acid hydrogels exhibit a minimal ratio of red blood cell destruction, indicating their low haemolytic activity. Beyond 94 hours, ferulic acid hydrogel demonstrates minimal toxicity towards human fibroblasts, suggesting it has good biocompatibility. When clinical parameters were compared after three months of treatment with SRP alone, significant reductions were observed in all parameters. However, when hydrogel application was done along with SRP, greater reduction was seen in terms of all clinical parameters indicating the efficacy of the ferulic acid hydrogel as an adjunct.

Conclusion

Ferulic acid has distinct haemolytic activity as well as good biocompatibility. Its use also led to a considerable reduction in all clinical parameters, necessitating its role as a local drug delivery agent in the treatment of periodontitis.

## Introduction

Periodontitis, a chronic multifactorial inflammatory disease, impacts the tissues supporting teeth, causing their progressive destruction. This condition results from changes in the bacteria within periodontal pockets and a varied host immune response. Exacerbating factors include systemic illnesses like diabetes mellitus or habits like smoking. This prevalent condition affects roughly 10-15% of the global population, according to estimates from the World Health Organization (WHO). Given its varied causes, tailored treatment approaches are essential, primarily focused on controlling the patient's infection. Supra- and subgingival instrumentation, commonly referred to as scaling and root planing (SRP), along with diligent home dental care, have an important role in managing the infection in patients diagnosed with periodontitis. These procedures target and eliminate the pathogenic bacteria, which organize themselves into biofilms and serve as the primary cause of periodontitis. Beyond mechanically eradicating the biofilm, additional host-modulating agents can aid in the treatment of periodontitis [1].

To optimize the effectiveness of pharmacological therapy while minimizing risks, controlling the release of the drug is crucial. That's why a local drug delivery system (LDDS) is recommended in combination with SRP. LDDSs show promise in various medical areas, treating localized infections in various body parts [2]. When managing periodontitis, maintaining a consistently effective concentration of the drug within the pockets for an adequate duration is vital [3]. Therefore, an LDDS serves as a valuable tool for adjunctive local pharmacological therapy in periodontal treatment. LDDSs regulate the discharge of locally administered drugs meant to supplement periodontal treatment. These systems fall into the following two categories: LDDSs loaded with therapeutic agents employed as adjuncts in non-surgical periodontal therapy and drug-loaded LDDSs utilized as adjuncts in surgical therapy. Treating periodontitis through local administration is preferred over systemic administration for several reasons. Systemic administration often leads to gastrointestinal problems, necessitates frequent dosages of drugs to sustain the drug in higher concentrations in the blood, and can result in additional side effects like dysbiosis or drug resistance [4].

LDDSs are available in various forms such as fibers, strips, films, microparticles, nanosystems, gels, etc. Among these, gels are a very popular option to be used in the field of dentistry as these systems boast high biocompatibility and bioadhesiveness, making them easily fabricated and administered. They are typically placed into the periodontal site using wide-port needle syringes. Natural products have been extensively studied and utilized in the creation of novel drugs for a considerable time [5]. Ferulic acid (FA), also known as 4-hydroxy-3-methoxycinnamic acid, is among the phenolic acids mainly identified in medicinal plants such as ginseng and certain Chinese teas. FA predominantly exists within plant cell walls and plays a crucial role in enhancing structural integrity and rigidity. It achieves this by forming covalent bonds with polysaccharides like arabinoxylans, which, in turn, act as precursors to lignin. A complex polymer, lignin gives mechanical strength and shields plant tissues from biodegradation [6]. Research has demonstrated the diverse pharmacological effects of FA and its derivatives, notably their antioxidative, anti-inflammatory, anti-cancer, and anti-fibrotic properties [7].

The term oxidative stress refers to situations where heightened levels of reactive oxygen species (ROS) are present [8]. Although its involvement in periodontitis is well-established, the concept of oxidative stress extends to elucidate the correlation between periodontitis and various systemic conditions such as diabetes mellitus and rheumatoid arthritis. Intriguingly, all these linked systemic conditions have treatments centered around the use of antioxidants to manage or mitigate the diseases. In the realm of periodontology itself, the impact of smoking [9], the pathogenesis of *Porphyromonas gingivalis*, and the potential cytotoxic effects of chlorhexidine digluconate have been expounded by applying the principles underlying oxidative stress. Thus, within the literature, diverse hydrogels have been employed alongside SRP. The results of many investigations have demonstrated that hydrogels possess the ability for controlled drug release and aid in periodontal wound healing. Hence, this study aimed to develop an FA hydrogel and assess its effectiveness for managing periodontitis.

## Materials and methods

FA hydrogel preparation

**Figure 1:**
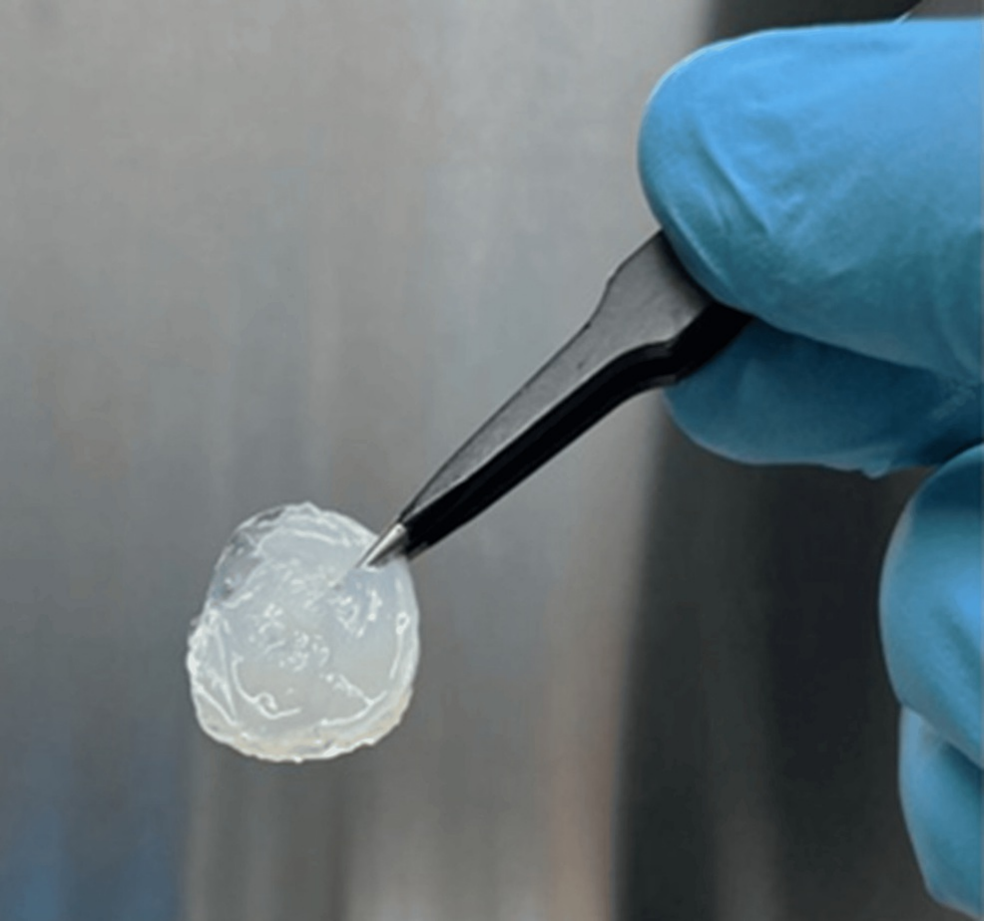
Prepared FA hydrogel FA: ferulic acid

A hydrochloric acid at a concentration of 1.5% weight/volume (w/v) and a separate solution of polyethylene glycol (PEG) 1 g/ml were prepared by dissolving 2 grams of each in 100 mL distilled water. These solutions were then combined at a ratio of 2:1, respectively, to create a hydrogel solution. The mixture was stirred every 10 minutes until a uniform consistency was achieved over a period of two hours. Following this, lithium phenyl-2,4,6-trimethylbenzoylphosphinate was added to create a 1:1 mixture. To this, 50 microgram/ml FA was added drop by drop. The mixed solution was then added to 0.145 M calcium chloride for ion cross-linking. Subsequently, a 10% volume/volume (v/v) ammonium hydroxide solution was added for 30 minutes in sequence. For the removal of residue, thorough washing with phosphate-buffered saline (PBS) was carried out, and bulk hybrid gels were fully swollen in PBS solution for 24 hours at a temperature of 37°C. The prepared hydrogel is depicted in Figure 1.

In vitro analysis

Haemolysis Assay

A diluted solution of whole blood was prepared by mixing 4 mL ethylenediaminetetraacetic acid (EDTA) whole blood with 5 mL of 0.9 wt% sodium chloride (NaCl). A 50 μL sample of this mixture was then added to 950 μL of 1× PBS solution and incubated for 30 minutes. Following this, 0.2 mL of diluted whole blood was added and further incubated at 37°C for one hour. Centrifugation was done at 1000 rpm for 10 minutes.

Biocompatibility Assay

The biocompatibility of the FA hydrogel was assessed using a 3-(4,5-dimethylthiazol-2-yl)-2,5-diphenyltetrazolium bromide (MTT) assay on human periodontal ligament tissue fibroblasts. Different percentages of the FA hydrogel were incubated for 24 hours. After incubation, cell viability was determined by replacing the medium with 10 μL of stock MTT dye (10 mg/ml) in each well, followed by incubation at 37°C for four hours. 

Study protocol

Twenty patients were chosen at random from the Department of Periodontics of Saveetha Dental College and Hospitals and assigned to one of the two groups. All patients were diagnosed with stage I grade A periodontitis, and each had a minimum of 20 natural teeth. All patients were between 25 and 55 years old. In the control group (Group A=10), only SRP was performed. In the test group (Group B=10), SRP followed by placement of FA hydrogel was carried out. Patients who had been on antibiotics for the past three months, those with systemic conditions, women who were pregnant or lactating women, and smokers were not included. The Institutional Review and Ethical Committee of Saveetha Dental College and Hospitals approved the study (approval number: IHEC/SDC/PERIO-2105/23/054). 

Informed consent was obtained from all participants. The sample size of 20, with a power of 80% and an alpha error of 95% confidence level, was determined. The clinical parameters assessed included pocket depth (PD), clinical attachment loss (CAL), plaque index (PI), and gingival index (GI), recorded before the start of treatment and after three months.

Periodontal therapy

SRP was performed. In addition, patients in Group B were treated with FA hydrogel. The hydrogel was administered into the pocket using a syringe equipped with a blunt cannula for subgingival delivery. Periodontal dressings were applied in both groups, which were removed after a week. Follow-up reviews were scheduled after three months.

Statistical analysis

Intergroup comparisons, evaluating SRP alone versus SRP with hydrogel, were performed using the independent t-test. Intragroup comparisons between baseline and three months were carried out using the paired t-test, and p<0.05 was considered statistically significant.

## Results

Haemolysis assay

**Figure 2:**
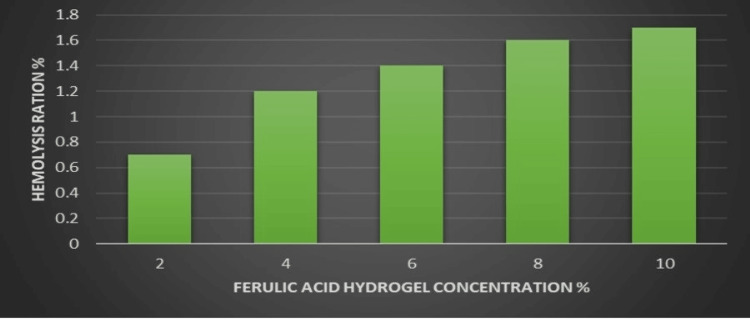
Haemolysis assay depicting the ratio of red blood cell destruction

At a concentration of 2%, the haemolysis ratio was <1%, while at concentrations of 4%, 6%, 8%, and 10%, the haemolysis ratio varied from 1.2% to 1.8%. These results suggest that FA hydrogels exhibit a minimal ratio of red blood cell destruction, indicating their low haemolytic activity, as depicted in Figure 2.

Biocompatibility assay

**Figure 3:**
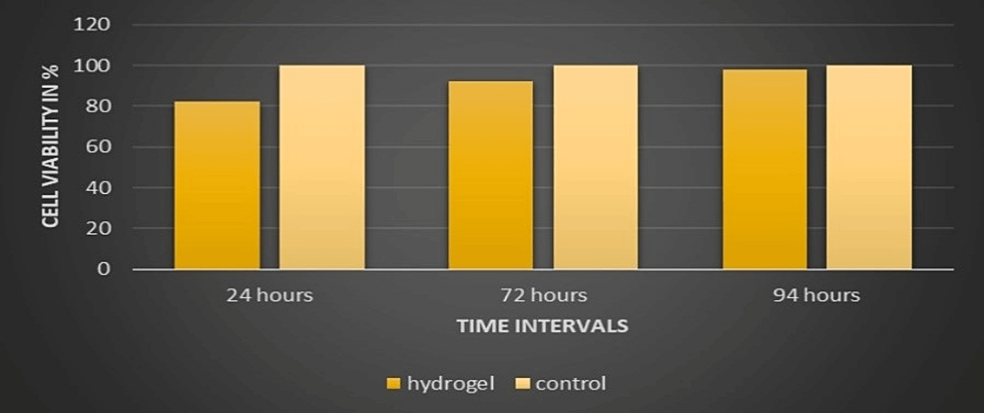
Biocompatibility assay comparing FA hydrogel with control FA: ferulic acid

Fibroblasts were exposed to varying concentrations of FA hydrogel for durations of 24, 72, and 94 hours. Subsequently, cell viability was evaluated using the MTT assay, with the data normalized to unexposed cells. Notably, after 24 hours of exposure, cell viability decreased to approximately 82% in the presence of the hydrogel. However, cell viability increased to 92% at 72 hours and further rose to 98% after 94 hours of exposure. These findings indicate that beyond 94 hours, FA hydrogel demonstrates minimal toxicity towards human fibroblasts, suggesting its biocompatibility, as depicted in Figure 3.

**Figure 4:**
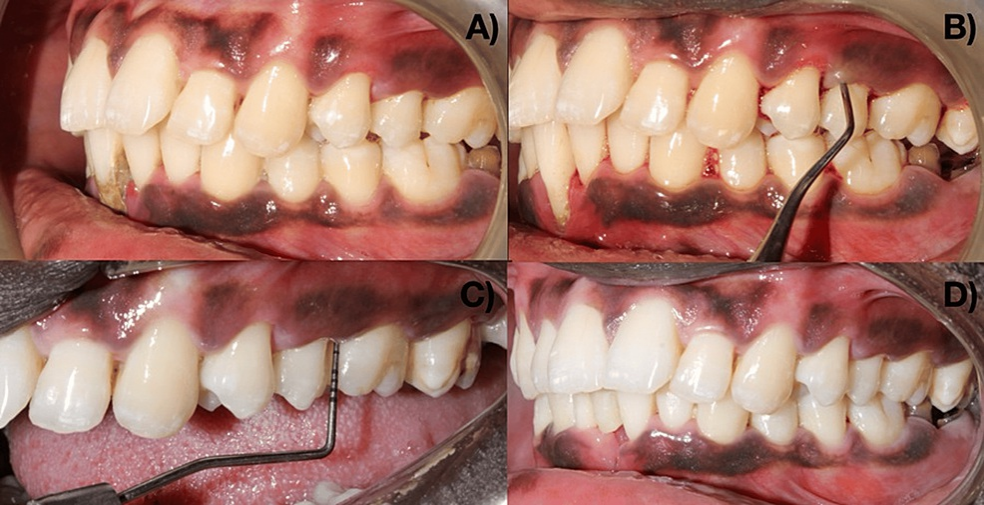
The prepared FA hydrogel was used in patients with moderate periodontitis (A) Preoperative view. (B) SRP performed. (C) Reduction in probing pocket depth after FA local drug delivery. (D) Postoperative view FA: ferulic acid; SRP: scaling and root planing

The hydrogel was used in patients with moderate periodontitis as depicted in Figure 4.

Here are the summarized demographic and clinical characteristics of both groups as presented in Table 1.

**Table 1:** The summarized demographic and clinical characteristics of both groups SRP: scaling and root planing

Parameters	Group A=SRP (mean±SD)	Group B=SRP+gel (mean±SD)	P-value
Age	35.40±2.27	37.59±2.55	0.2
Gender (male/female)	7/3	5/5	0.27
Probing index	2.37±0.59	2.47±0.27	0.44
Gingival index	2.47±0.22	2.73±0.80	0.56
Probing pocket depth	5.09±0.78	5.43±0.88	0.69
Clinical attachment loss	3.06±0.67	3.12±0.61	0.71

Independent t-tests to compare baseline values and three-month values between the two groups were done. The baseline values showed no significant difference, with p-values greater than 0.05, as described in Table 2.

**Table 2:** Comparison of baseline values between the two groups

Parameters	Group A	Group B	P-value
Plaque index	2.37±0.59	2.47±0.27	0.44
Gingival index	2.47±0.22	2.73±0.80	0.56
Probing pocket depth	5.09±0.78	5.43±0.88	0.69
Clinical attachment loss	3.06±0.67	3.12±0.61	0.71

After three months, all parameters showed a significant difference as seen in Table 3. 

**Table 3:** Comparison of three-month values between the two groups *statistically significant

Parameters	Group A	Group B	P-value
Plaque index	1.79±0.57	0.45±0.10	0.00*
Gingival index	1.48±0.53	0.88±0.51	0.00*
Probing pocket depth	3.78±0.50	3.01±0.44	0.00*
Clinical attachment loss	2.03±0.58	1.98±0.55	0.00*

After three months, a higher reduction in all parameters was seen in Group B. A p-value of 0.00 was obtained for all parameters. This indicates that SRP along with hydrogel application led to better clinical outcomes. A paired t-test was used for comparison of the baseline values and values recorded after three months for Groups A and B, described as follows in Table 4.

**Table 4:** Comparison of the baseline values and values recorded after three months for Groups A and B *statistically significant

Variables	Group A (mean±SD)			Group B (mean±SD)		
	Baseline	Three months	P-value	Baseline	Three months	P-value
Probing index	2.37±0.59	1.79±0.57	0.00*	2.47±0.27	0.45±0.10	0.00*
Gingival index	2.47±0.22	1.48±0.53	0.00*	2.73±0.80	0.88±0.51	0.00*
Probing pocket depth	5.09±0.78	3.78±0.50	0.00*	5.43±0.88	3.01±0.44	0.00*
Clinical attachment loss	3.06±0.67	2.03±0.58	0.00*	3.12±0.61	1.98±0.55	0.00*

After three months of treatment with SRP alone, significant reductions were observed in all parameters. Specifically, the PI decreased from 2.37±0.59 mm to 1.79±0.57 mm, the GI decreased from 2.47±0.22 mm to 1.48±0.53 mm, the probing PD decreased from 5.09±0.78 mm to 3.78±0.50 mm, and the CAL decreased from 3.06±0.67 mm to 2.03±0.58 mm. The p-values for all parameters were less than 0.05, indicating a statistically significant difference.

## Discussion

In recent times, local drug delivery has become a focal point in the treatment of periodontitis, garnering both significant attention and research efforts. This approach facilitates precise and controlled delivery of medicines by directly administering therapeutic agents to the affected sites. Evidence from the literature indicates that FA exhibits sensitivity towards aerobes and anaerobes and it also effectively inhibits biofilm formation [10]. The present study aimed to develop, characterize, and evaluate the clinical effectiveness of FA-based hydrogel as an LDDS for treating periodontitis. Literature suggests that hydrogels can provide sustained drug release while also providing targeted drug delivery to affected periodontal tissue areas [11]. In the present study, FA hydrogel was synthesized, followed by haemolytic analysis and biocompatibility testing. Results indicated a low ratio of red blood cell destruction and deemed the FA hydrogel as a significantly non-toxic substance.

The preparation and properties of several hydrogels, such as polyvinyl alcohol (PVA), sodium alginate, and BAG hydrogels, exhibited superior haemostatic properties and biocompatibility [12]. Additionally, another study focused on preparing in situ linezolid gel and evaluating its appearance, viscosity, pH, and drug release pattern. These studies contribute valuable insights into the development and characterization of hydrogels for local drug delivery applications [13]. Previous literature demonstrated that FA hydrogel exhibits favorable biocompatibility properties and improved haemostatic capabilities compared to conventional hydrogels [14].

Additionally, on evaluation of the efficacy of FA hydrogel, patients treated with SRP along with hydrogel exhibited significant reductions in clinical parameters. In a similar study, 2% minocycline was utilized as a local medication delivery method for periodontitis patients, resulting in notable decreases in probing PD in both groups over six and 12 weeks. However, the findings of this study did not show a statistically significant difference [15]. Similarly, another study showed that using 1% alendronate gel led to significant reductions in probing PD and CAL at two- and six-month intervals [16]. In a similar study, 3% satranidazole gel was employed along with SRP, and it was concluded that there was a reduction in probing PD as well as CAL [17]. Our findings align with these previous studies, highlighting the efficacy of LDDSs in improving periodontal parameters.

Limitations

The in vitro study in this research lacks analysis of the release pattern and the rate of degradation of the hydrogel. Additionally, the study had a limited sample size. To further validate the findings and ensure robustness, a randomized controlled trial with a larger sample size is warranted. This would provide more comprehensive data and strengthen the evidence supporting the efficacy and safety of the FA hydrogel in the treatment of periodontitis. 

## Conclusions

FA, because of its distinct haemolytic activity and good biocompatibility, can be a potential candidate for use in periodontal therapy. Along with this, it also displays considerable reduction in all clinical parameters such as probing PD, CAL, PI, and GI, indicating the importance of its role as a local drug delivery agent in the treatment of periodontitis.
